# The influence of gastrointestinal pH on speciation of copper in simulated digestive juice

**DOI:** 10.1002/fsn3.2490

**Published:** 2021-07-26

**Authors:** Min Wu, Leqin Ke, Mingyu Zhi, Yumei Qin, Jianzhong Han

**Affiliations:** ^1^ Hangzhou Vocational & Technical College Ecology and Health Institute Hangzhou China; ^2^ Food Safety Key Laboratory of Zhejiang Province School of Food Science and Biotechnology Zhejiang Gongshang University Hangzhou China

**Keywords:** copper, digestion, pH, simulated digestive juice, speciation

## Abstract

Speciation can provide knowledge about absorption, reactivity to binding sites, bioavailability, toxicity, and excretion of elements. In this study, the speciation of copper in different model solutions under the influence of gastrointestinal (GI) pH was studied by ion selective electrode (ISE) and inductively coupled plasma optical emission spectrometry (ICP OES). It was found that the electrode response (mV) against Cu^2+^ decreased with the increase in pH and dropped to the lowest point at pH 7.5 in all model solutions. When amino acids and organic acids were present, the ratio of filtered copper (0.45 μm, pH 7.5) was more than 90%. When casein was present, whey protein, pancreatin, and starch were added, and the ratio of filtered copper was 85.6 ± 0.3, 56.7 ± 8.8, 38.5 ± 5.1, and 1.0 ± 0.3%, respectively. When there is not enough organic ligand, excessive copper will form copper hydroxide precipitation with the increase in pH, but it got the highest electrode response (mV) against Cu^2+^. From this study, it can be concluded that the speciation of copper in GI tract is strongly influenced by the pH and the composition of food. When there are few ligands coexisting in the GI tract, the concentration of copper ion may be relatively high.

## Graphical abstract

The speciation of copper with different food components/digestive juice components in the environment of digestive tract was studied by ion selective electrode and inductively coupled plasma optical emission spectrometry. The result indicates that different types of food will lead to different forms of copper distribution. The food rich in soluble ligands may lead to high soluble copper and then high bioavailability. And the food without ligands (water) may have high copper ions in digestive tract, which may lead to toxicity.

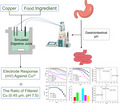

## 1 INTRODUCTION

2003Copper is an essential trace metal with a crucial role in various biological systems. As a structural or catalytic cofactor, it was required for respiration, connective tissue formation, iron metabolism, and many other processes (Zehra et al., 2021). However, excessive copper intake or copper excretion disorders can lead to oxidative tissue damage through free radical‐mediated pathways, resulting in toxicity, such as Indian childhood cirrhosis and Wilson's disease (Dai et al., 2020; Gaetke et al., 2014; Jomova & Valko, 2011; Pierson et al., 2019). The imbalance of copper also promotes the production of neurodegenerative diseases (Li & Kerman, 2021; Ma et al., 2020; Pal, 2014; Squitti et al., 2014). Therefore, the balance of copper is important to human health.

The major source of copper is food for humans, and the absorption and toxicity of copper in various food seem different. Organic copper has higher bioavailability and lower toxicity than inorganic copper (Liu et al., 2019; Vieira et al., 2020; Zhi et al., 2020). The protein source had a significant effect on copper bioavailability in rainbow trout; those fed diet with soy and corn proteins require lower copper than those fed menhaden and blood proteins (Read et al., 2014). The efficiency of apparent copper absorption from the lacto‐ovo‐vegetarian diet was less (33%) than that from the nonvegetarian diet (42%) (pooled *SD*: 9%; *p* < .05) (Hunt & Vanderpool, 2001). The food with different composition (breakfast, lunch, and dinner) leads to various copper bioaccessibility (8.48 ± 3.54, 27.04 ± 10.49, and 31.09 ± 18.08%, respectively) (Velasco‐Reynold et al., 2007). In our previous study, the mice that intake copper with water have higher oxidative stress, serum‐free copper, and brain's Aβ_1‐42_ when compared to mice intake copper with food (Wu et al., 2016).

The different absorption rate and toxicity of copper in various food are related to its speciation, such as the concentration of dissolved copper, copper ions, and binding strength of ligands with copper in the lumen of the GI tract. Despite the complex and variable environments during digestion, previous research has established that the pH gradient in the GI tract is one of the most important environmental factors for the speciation of copper (Dendougui & Schwedt, 2002). Few researches are focused on the changes in speciation of copper in the process of digestion. Mills add copper in an aqueous extract of herbage and found copper was in the form of complexes that appear to be stable above pH 2–5 (Mills, 1956). Schwedt uses ion selective electrode (ISE) to determine the complexing of copper in food extracts and predicted that all the copper absorbed by intestines will be complexed, after neutralization no free ions can be found (Dendougui & Schwedt, 2002). Although there have been some achievements in the changes of copper during the pH gradient in the GI tract, it is also difficult to deduction the solubility and complexation of copper in the digestive tract, especially for different types of food.

To gain a better understanding of the copper speciation in humans’ GI tract, copper and some food components/digestion enzymes were added in the simulated gastric fluid (SGF). The electrode response (mV) against Cu^2+^, which is linearly related to the logarithm of the Cu^2+^ concentration under standard conditions (Momin & Pillai, 2015; Silanikove et al., 2003), was tested at a pH of 2.0–7.5 by ISEs. The ratio of dissolved copper, which was defined as that which passes a membrane filter with 0.45 μm pore size (Stella & Ganzerli‐Valentini, 1980; Stiff, 1971), was tested by ICP‐OES.

## 2 MATERIALS AND METHODS

### 2.1 Apparatus

The pH measurements were done using Titrando 902 with a pH electrode (Metrohm, Swiss) and software tiamo 2.4. The NaOH solution was added automatically by 800 Dosino (Metrohm, Swiss) equipped with a 20‐ml burette. The potential was measured by SevenExcellence^TM^ (Mettler Tolido, Swiss) with software LabX direct pH 2.4, which can automatically record the potential at an interval of 1 s. A copper ion selective electrode (Bante, U.S.A.) was used to measure potential variation. A magnetic stirrer (IKA, Germany) and a constant temperature water bath apparatus (Guohua Electronic Appliance Co., Ltd., China) were used.

Copper concentration was determined by ICP OES (SPS8000 Bjhaiguang Co., Ltd,) under standard operating conditions.

### 2.2 Reagents

Ultrapure water (Thermo science® NANOpure, U.S.A) was used. Copper (II) sulphate‐5‐hydrate (99%–102%), oxalic acid (≥98%), citric acid monohydrate (≥98%), DL‐malic acid (≥98%), ethylene diamine tetra acetic acid (EDTA, ≥98%), L‐histidine (≥98%), L‐glutamic acid (≥99%), L‐tyrosine (≥99%), glycine (≥98%), D‐(+)‐glucose (≥99.5%), sucrose (≥99%), D‐(‐)‐fructose (≥99%), starch (from wheat), casein (Hammarsten bovine, protein >95%), pepsin (pepsin from porcine gastric mucosa, ≥250 unites m/g solid), and pancreatin (pancreatin from porcine, 4×USP specifications) were all purchased from Sigma Aldrich. Whey protein (food grade) was purchased from Ji'nan Shenghe Chemical Co., Ltd; bile was extracted from fresh porcine gall bladders and stored at −20°C until use.

Commercially available standard solution of Cu (1,000 mg/L; Institute for Environmental Reference Materials of Ministry of Environmental Protection [IERM], China) was used to prepare calibration curves.

### 2.3 Preparation of the model solutions

Before experiment, the composition of simulated gastric fluid (SGF, 1.25×concentrates) was determined as previously described (Minekus et al., 2014), and copper stock solution of accurate concentration was prepared. In 100 ml ultrapure water, 4.69 g CuSO_4_.5H_2_O was dissolved, and the accurate concentration was tested by ICP OES.

Divided the Food/digestion components are into three groups. The components that molecular structure are known (oxalic acid, citric acid monohydrate, DL‐malic acid, EDTA, L‐histidine, L‐glutamic acid, L‐tyrosine, glycine, D‐(+)‐glucose, sucrose, and D‐(‐)‐fructose) were defined as known structure ligands group (KSLG), and the food components that structure are complex (starch, casein, and whey protein) were defined as complex ligands groups (CLG), while the pepsin, pancreatin, and bile were digestive ligands group (DLG).

Blank solution: Added 64‐ml SGF solutions (1.25×concentrates) and 1.00‐ml copper stock solution, after adjusting the pH to 2.0 with HCl solution, to make up the volume to 80.0‐ml ultrapure water. The concentration of copper in the model solution was around 150 mg/L.

KSLG groups: Added the reserve solution according to the above method, and then 31.25 mmol/L food components that were 10 times of copper were added.

CLG groups: Added the reserve solution according to the above method, and then 0.80 g protein or starch was added.

DLG groups: The reserve solution was added according to the above method, and then 0.256 g of pepsin/pancreatin was added in the solution. The concentration was according to the in vitro digestive model previously described (3.2 g/L)(Liu et al., 2012, 2013). Add 5.5‐ml fresh bile to make the bile salt 10 mmol/L in the solution (Minekus et al., 2014). The concentration of the bile salt was measured using commercial assay kits (Jiancheng Institute, Nanjing, China).

### 2.4 Potential measurements

The pH electrode and ISE were immersed in the solution before titration. After mixing for 5 min, sodium hydroxide solution was slowly added by Titrando to raise the pH from 2.0 to 7.5. The data (pH and electrode responses against Cu^2+^) were automatically recording by tiamo 2.4. and LabX direct pH 2.4. During the titration process, a 37°C water bath, a constant electrodes distance, and stirring speed were offered.

pH and copper ion selective electrodes were checked every day before the measurement, and the membrane was polished with aluminum hydroxide when necessary.

### 2.5 Percentage of dissolved copper

The added volume was record by Titrando 902 and the solution was filtrated with MF^TM^ Membrane Filters (0.45 μm, Merck Millipore Ltd.). The concentration of filtered copper was tested by ICP OES under standard operating conditions. Quality control samples were analyzed between every five samples. Calibrations were based on 2.0 M nitro acid. Equation 1 was used to calculate the dissolved ratio of copper.

$$\text{Dissolved}\;\text{ratio}\;\text{of}\;\text{copper}=\frac{C_{\text{Filtered}\;\text{Cu}}\times \left(\text{Initial}\;\text{volume}+\text{Added}\;\text{volume}\right)}{m_{\text{Total}\;\text{Cu}}}\times 100\%\left(1\right)$$

C_Filtered Cu_—The concentration of filtered copper tested by ICP OES.

m Total Cu—Total amount of copper added in the solution (around 12 mg), which was verified by ICP OES.

Initial volume—80.0 ml.

Added volume—record by Titrando 902.

### 2.6 Statistical analysis

The experiments were performed in triplicate, and experimental data are expressed as the mean ± standard deviation (*SD*). Statistical significance was determined by one‐way ANOVA using IBM SPSS Statistics version 22 (IBM Corporation, New York, U.S.A.). A probability of *p* < .05 was considered statistically significant.

## 3 RESULTS

### 3.1 Dissolved copper and electrode response (mV) against Cu2+ in KSLG

**FIGURE 1:**
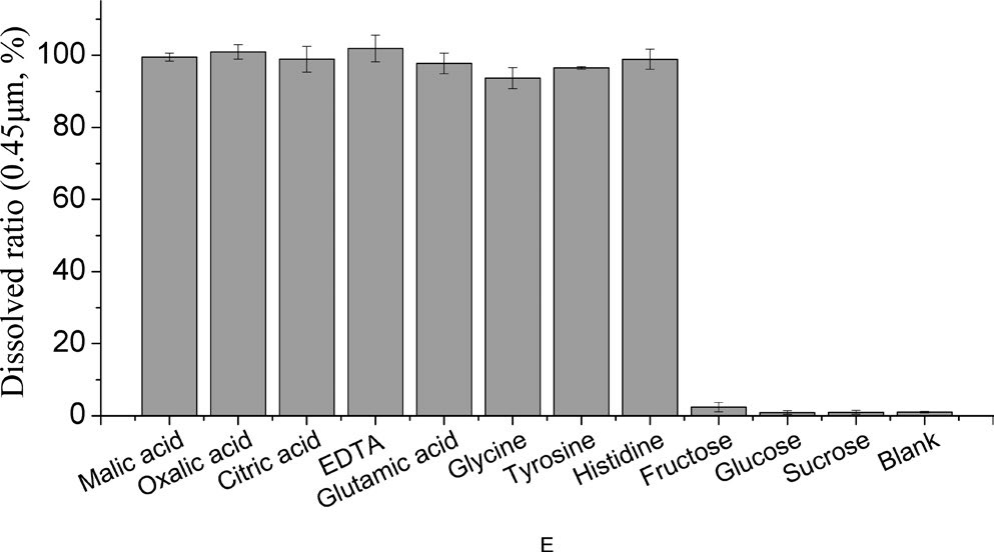
Ratio of dissolved copper in KLSG, *n* = 3

It can be observed that some flocculent precipitates were appeared along with the raise of pH in the blank, fructose, glucose, and sucrose solutions. However, it has always been clear in organic acid and amino acid solutions. The results of Figure 1 and Table 1 showed that organic acids and amino acids model solutions were almost completely dissolved, while blank, fructose, glucose, and sucrose solutions rarely dissolved (the proportion of copper below 3%). The visible precipitate and low solubility of copper indicate the production of copper hydroxide precipitation in the blank, fructose, glucose, and sucrose solutions.

**TABLE 1:** Concentrations of dissolved copper in KLSG

Group	Dissolved concentration^a^ (0.45 μm, mg/L)	Group	Dissolved concentration^a^ (0.45 μm, mg/L)
Malic acid	80.8 ± 0.7	Tyrosine	126.3 ± 0.6
Oxalic acid	105.9 ± 2.0	Histidine	92.1 ± 2.2
Citric acid	79.3 ± 3.1	Fructose	5.2 ± 2.3
EDTA	76.1 ± 2.7	Glucose	3.9 ± 1.5
Glutamic acid	93.5 ± 2.3	Sucrose	1.3 ± 0.6
Glycine	113.8 ± 3.2	Blank	1.0 ± 0.2

^a^ Due to the different amount of sodium hydroxide solution added in these solutions, these final total volumes were different.

**FIGURE 2:**
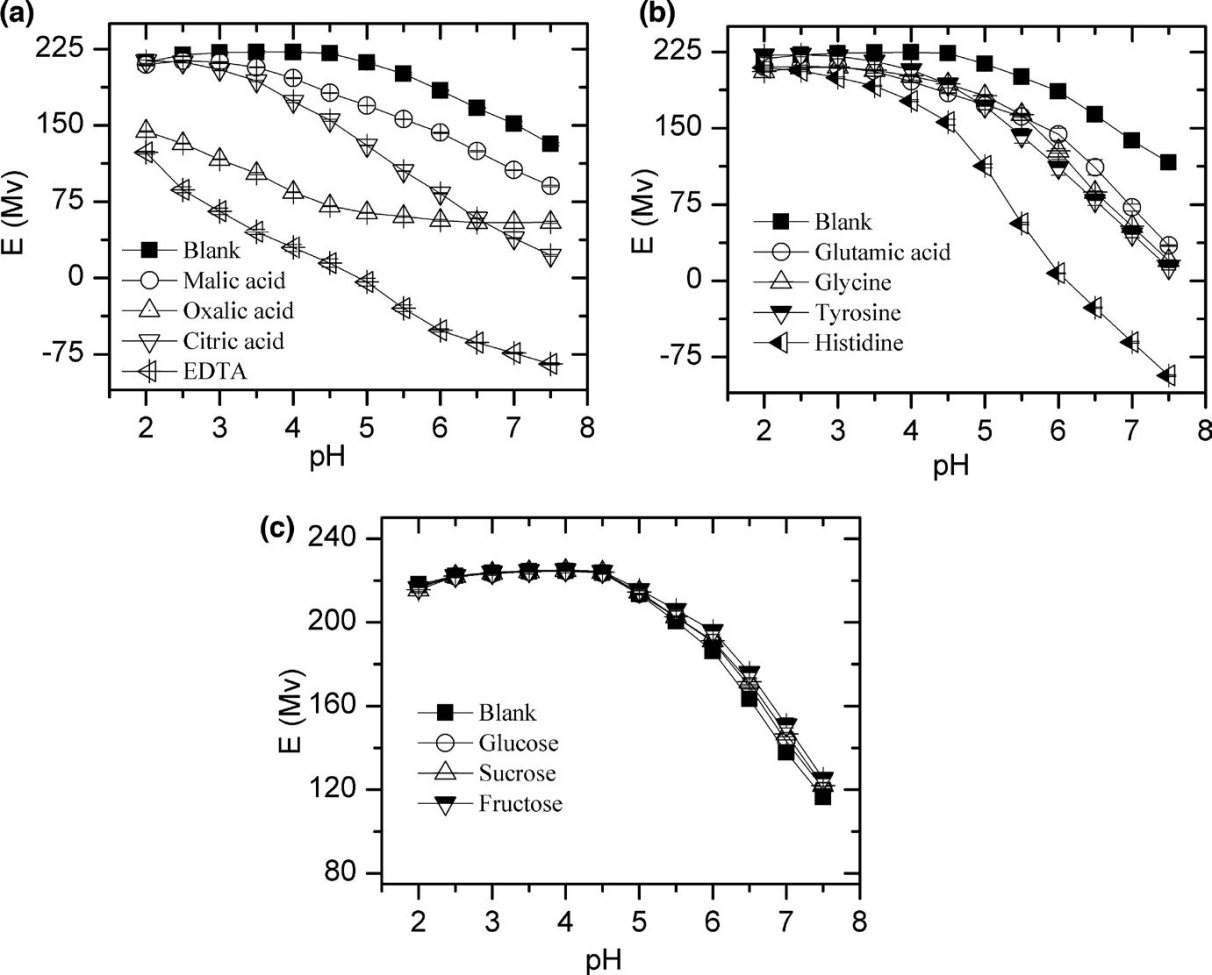
Electrode response (mV) in organic acid (a), amino acid (b), and simple carbohydrates (c) model solutions between pH 2.0 and 7.5, *n* = 3

Figure 2 presents the changes in electrode response (mV) against Cu^2+^ with the increasing pH. The responses were high during pH 2–4 in most of these model solutions (187–224 Mv). With the proceeded increase in pH, the potential decreased and dropped to the lowest point at pH 7.5. As can be seen from the Figure 2c, the electric potentials changes in fructose, glucose, and sucrose solutions are extremely similar with blank solution, which further confirmed the formation of copper hydroxide precipitation. Compared to blank and simple carbohydrates solutions, the change in electrode response in organic acid and amino acid solutions was more obvious (Figure 2a, Table 2). Except for oxalic acid solution, the ΔE_pH 2.0‐7.5_ of organic acid and amino acid solutions were all above 119 Mv (Figure 2b). Considering the higher solubility of copper and lower potential of Cu^2+^ in these solutions, we can see that these organic acids or amino acids can form soluble complexes with copper, which increase the solubility of copper and decrease the content of copper ions.

**TABLE 2:** The change in electrode response in KLSG

Group	ΔE_pH 2.0−7.5_ (Mv)	Group	ΔE_pH 2.0−7.5_ (Mv)
Malic acid	119.2 ± 1.9	Tyrosine	192.3 ± 1.0
Oxalic acid	89.3 ± 0.6	Histidine	302.5 ± 1.4
Citric acid	191.2 ± 3.9	Fructose	68.7 ± 1.0
EDTA	207.8 ± 1.3	Glucose	77.1 ± 0.9
Glutamic acid	175.5 ± 2.0	Sucrose	74.9 ± 1.4
Glycine	185.8 ± 0.3	Blank	80.4 ± 1.7

In contrast to other model solutions, the electrode response was lower at pH 2.0 in oxalic acid (144.1 ± 0.7 Mv) and EDTA (123.2 ± 1.5 Mv) model solutions (Figure 1a). The change in electrode response was slow after pH 6.0 for oxalic acid solution (56.7 ± 0.8 Mv at pH 6 and 54.8 ± 1.0 Mv at pH 7.5), while the potential variation was still obviously decreased for the EDTA solution (−51.6 ± 1.6 Mv at pH 6 and −84.5 ± 1.1 Mv at pH 7.5) and other solutions. The initial potential change indicates that oxalic acid and EDTA begin to form complex with copper at pH 2, with the continued increase in pH, the complexation of oxalic acid with copper reached saturation, while the complexation between EDTA and copper did not (Figure 1a).

### 3.2 Dissolved copper and electrode response (mV) against Cu2+ in CLG and DLG

**FIGURE 3:**
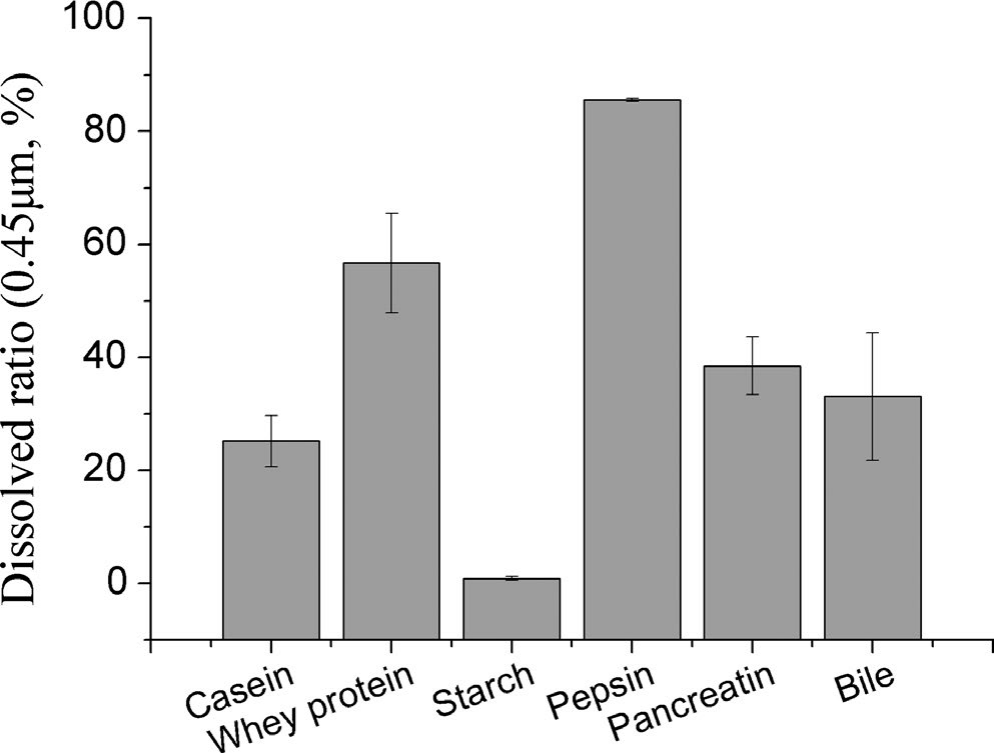
Ratio of dissolved copper in CLG and DLG, *n* = 3

The dissolved ratios in CLG and DLG were different from each other (Figure 3, Table 3). The ratio in casein, whey protein, and pancreatin solutions was 85.6 ± 0.3, 56.7 ± 8.8, 38.5 ± 5.1%, respectively. Little copper existed in dissolved form (1.0 ± 0.3%) in starch model solution.

**TABLE 3:** Concentrations of dissolved copper in CLG and DLG

Group	Dissolved concentration^a^ (0.45 μm, mg/L)	Group	Dissolved concentration^a^ (0.45 μm, mg/L)
Casein	35.7 ± 6.3	Pepsin	127.2 ± 0.3
Whey protein	83.2 ± 13.0	Pancreatin	53.8 ± 7.2
Starch	1.0 ± 0.3	Bile	50.5 ± 4.7

^a^ Due to the different amount of sodium hydroxide solution added in these solutions, these final total volumes were different.

**FIGURE 4:**
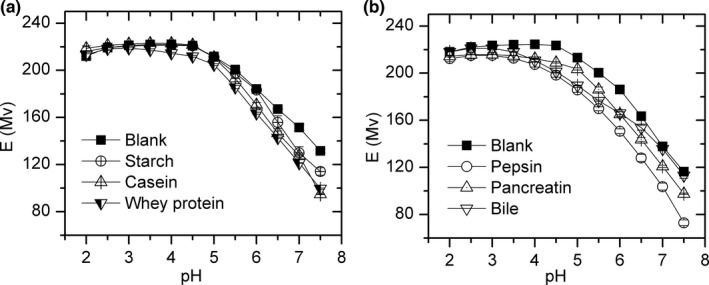
Electrode response (mV) in CLG (a) and DLG (b) model solutions between pH 2.0 and 7.5, *n* = 3

The electrode response changes in CLG and DLG (Figure 4) have the same trend as KLSG. All of them showed a high copper potential at low pH and decreased rapidly with the rise of pH, and reached the lowest value at pH 7.5. The ΔE_pH 2.0‐7.5_ of casein, pepsin, whey protein, starch, and pancreatin solutions was 149.5 ± 1.3, 139.4 ± 0.5, 113.9 ± 3.8, 102.0 ± 2.6, and 116.7 ± 0.3 Mv, respectively (Table 4). The ΔE_pH 2.0‐7.5_ and various solubility indicate copper complex with these ingredients, and the complex had different molecular size

**TABLE 4:** The change in electrode response in CLG and DLG

Group	ΔE_pH 2.0−7.5_ (Mv)	Group	ΔE_pH 2.0−7.5_ (Mv)
Casein	149.5 ± 1.3	Pepsin	139.4 ± 0.5
Whey protein	113.9 ± 3.8	Pancreatin	116.7 ± 0.3
Starch	102.0 ± 2.6	Bile	105.1 ± 2.3

## 4 DISCUSSION

According to the Nernst equation, an ISE produces a potential that is linearly related to the logarithm of the Cu^2+^ concentration under standard conditions (Momin & Pillai, 2015; Silanikove et al., 2003). In our study, although electrode drift may have occurred, this potential can still reflect the concentration of Cu^2+^ (De Marco et al., 2007; Momin & Pillai, 2015). The different electrode responses against Cu^2+^ for these model solutions indicate the concentrations of Cu^2+^ in digestive tract may be different in the presence of different ligands.

Trance metals in aqueous solutions can exist as free (hydrated) ions, complexes with organic or inorganic ligands. Considering the solubility product constant of cupric hydroxide (*K*_sp_ = 2.2 × 10^–20^) (Speight, 2005), most of the copper may bind to hydroxyl ions to form cupric hydroxide in the blank solution with the decrease in hydrogen ions, which is hard to dissolved in water, and the concentration of copper ions in pure water should be 0.014 mg/L at pH 7.5 under standard conditions. However, the results of the filtered copper concentration in blank solution were 1.0 ± 0.2 mg/L in our study, which is much higher than 0.014 mg/L. As there were only some inorganic ions existed in the blank solution, these ions may combine with copper to form copper complexes, which will affect the solubility of copper. The stability constant (lg*K*) was 4.31 with ammonia, 0.1 with chloride, and 6.79 with pyrophosphate (Speight, 2005). In another hand, some small precipitates may also pass through the membrane to increase the concentration of filtered copper. Same as the blank solution, low solubility and similar potential changes occurred in the fructose, glucose, and sucrose solutions (Figure 1c). Consider the protons are difficult to dissociate from these components, they are not good ligands for copper. Along with the rise of pH, flocculent precipitate was produced and the dissolved ratio decreased. From the study of these solution, it can be seen that the food without sufficient ligand may not provide enough binding sites for copper during digestion. Hence, with the decrease in pH, excess copper will be combined with hydroxyl to form copper hydroxide precipitation. However, although the dissolved copper concentration is low in these solutions, the electrical potential reflects that the content of copper ion in GI tract is much higher than other solution.

**FIGURE 5:**
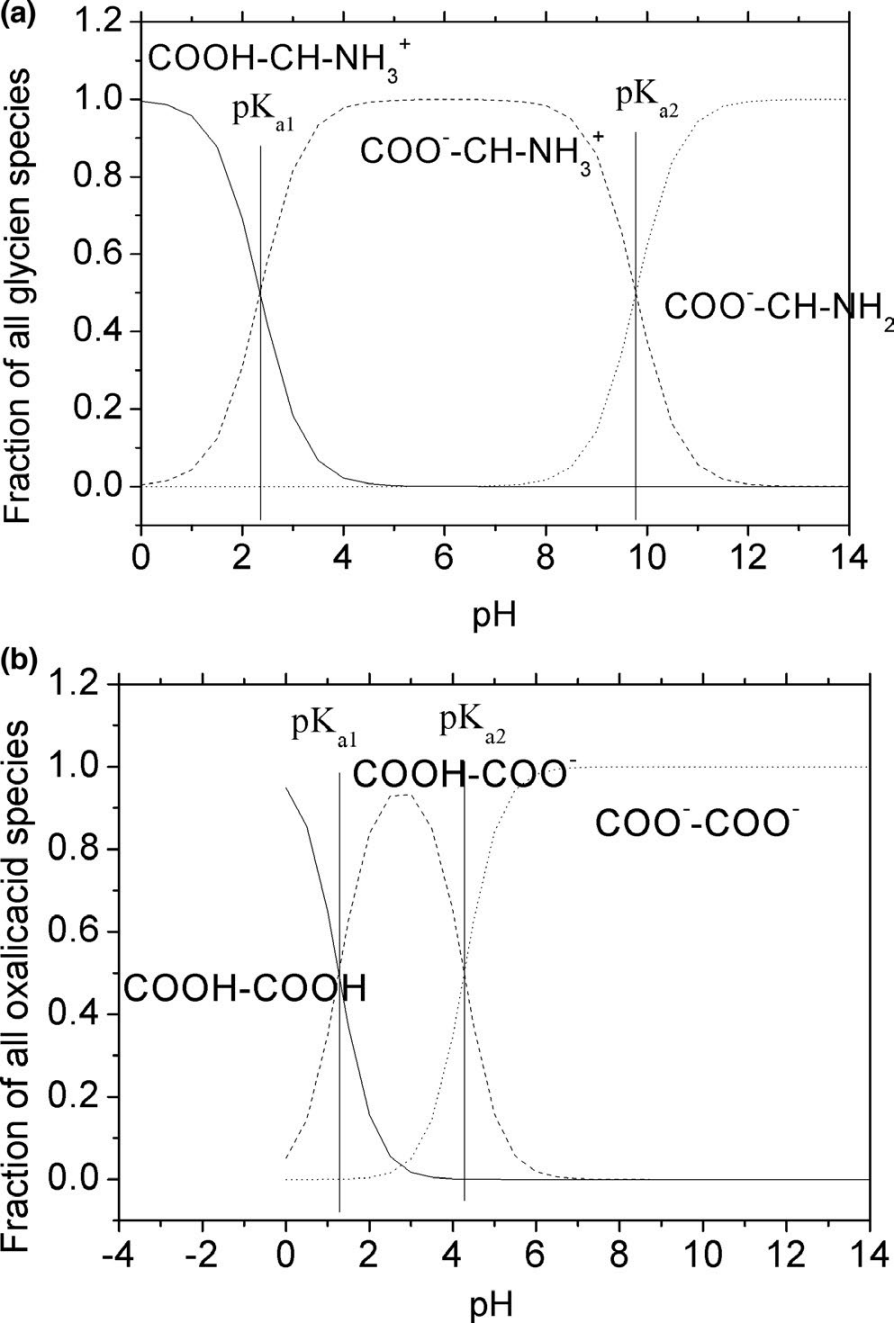
Fraction of all glycine species (a) and all oxalic acid species (b)

By contrast, N_am_, O_COO_, and nitrogen atoms of imidazole rings (N_im_) show remarkable affinities for copper (Carrera et al., 2004; Kang et al., 1985; Manceau & Matynia, 2010). The stability constant (lg*K*) of copper complexes was 4.35 and 14.2 with citric acid, 6.16 and 8.5 with oxalic acid, 8.60 and 15.54 with glycine, 4.35 (Cu_2_L) and 14.2 (Cu_3_L) with glutamic acid, 18.8 with EDTA, and 18.1 (CuL_2_) with histidine (Martell & Smith, 1974, 1989; Speight, 2005). Hence, organic acids and amino acids are good ligands for copper. With the raise of pH, they dissociate protons and offer binding sit for copper, copper complex with them to form stable and soluble complex, and lead to low copper potential and high dissolved ratio. Take glycine and oxalic acid as examples, fractions of all glycine species can be plotted using the acid–base dissociation constants (pKa) of glycine (Figure 5a). At low pH, the content of hydrogen ion is high and glycine tends to exist in the form of Lewis acid; and with the increase in pH, the concentration of hydrogen ions decreases, and glycine changes the morphology and formed the structure of Lewis base, which is easy to combine with copper to form soluble complex compound. The same change occurred in oxalic acid. However, as they have different pKa, the dissociation of ligands is different at the same pH value. At pH 2, there is already a large number of oxalic acids that exist in the form of COOH‐COO^‐^ (Figure 5b), which can complex with copper ions in acidic environment; and when the pH is above 6, two protons in oxalic acid are lost, the content of ligands did not increase, and the change in electrode response was slow after pH 6.0 for oxalic acid solution (56.7 ± 0.8 Mv at pH 6 and 54.8 ± 1.0 Mv at pH 7.5). For glycine, most of them are still in the form of COOH‐CH‐NH^3+^ at pH 2, which cannot complex with copper. At pH 6, glycine exists in the form of COO^‐^‐CH‐NH_3_^+^, and the amino group was not beginning to dissociate. Protons will continue to dissociate from glycine until pH 12. And the potential of glycine solution was still dropping rapidly above pH 6. The results in organic acids and amino acids solutions indicate that as the organic ligands have different acid–base dissociation constants, the complexation of ligands with copper is also different in the GI pH. In summary, the different changes in KSLG suggest that the speciation of copper in GI tract is closely related to the pH and the types of food. When there are few ligands coexisting in the GI tract, the concentration of copper ion may be relatively high.

The dissolved ratio of copper is variable in CLG and DLG groups. Copper combined with these components to form complexes, and decrease the concentration of copper ions, and the molecular size of these complex may have big size and cannot pass the pore of 0.45 μm. Starch molecules have long‐chain structures and are difficult to pass through the membrane; the dissolved ratio in starch solution is small. Casein may form micelles that are bigger than whey (Donato & Guyomarc'H, 2009), and the dissolved ratio of copper in casein is lower than whey protein. Pepsin and trypsin may continue to degrade into small molecules during the reaction, and their degradation products can also be complexed with copper. It is interesting to find that the potential reduction in protein solutions (between 102 and 150 Mv) was less than that of amino acids solutions (176 and 302 Mv). The stability constant (lg*K*) of copper complexes was 7.59 (ML/M.L), 4.4 (MHL/ML.H), and 16.68 (ML _2_ /M.L) with glycyl‐glycyl‐L‐histidine. However, its 18.1 (CuL_2_) with histidine (Speight, 2005; Martell & Smith, 1974; Martell & Smith, 1989). The different stability constant (lg*K*) of copper complexes indicates, although peptide and protein may offer lots of binding cites for copper, the molecular steric hindrance may hinder the binding of copper to ligands. Therefore, the complexation of protein with copper is also related to the molecular structure of protein. Fresh bile is complex mixture containing bilirubin, amino acids, bile salts, and other components (Hofmann & Mysels, 1992). The coordination between bile and copper was actually an instantaneous equilibrium state of various complex reactions and dissociation reactions. The dissolutive ratio of copper in bile model solution is 33.1%, which indicates that some kinds of large molecules can react with copper in bile and cannot pass through the membrane. The dissolved ratio and electrical potential of CLG and DLG groups indicate that the speciation of copper in digestive tract is very complex, and are affected by many factors. The species and concentration of coexisting ligands, their acid–base dissociation constants and stability constants with copper, the molecular steric of these ligands, and pH of intestinal tract, etc. All the factors will influence each other to achieve a microbalance.

Digesta is a complex mixture and the digestion process is dynamic; it is impossible to theoretically calculate the speciation of copper for the complex reaction mechanisms and unknown stability constants. However, the ligands in digesta may be numerous, ranging from low molecular weight compounds to large undefined macromolecules, and exhibit continuum binding strength; different types of food have different trends. The food rich in small ligands with strong binding strength may raise the solubility of copper, whereas the food rich in ligands that have large molecular size, strong binding strength, and hard to be degradation may have low copper bioaccessibility. This finding is consistent with that of Moreda‐Piñeiro (2012) who found a good negative correlations between the copper bioavailability percentage and the protein contention of the foodstuff (Moreda‐Piñeiro et al., 2012).

Another important finding was that the content of copper ion is different in various types of food. The concentration of copper ions may be extremely low in the presence of organic matter, and high when the ligand is insufficient. However, trace amount of Cu^2+^ has been detected in food samples, living zebrafish (Zhou et al., 2021), and AD rat brains (Yu et al., 2017) and significant toxicity was found. The toxicity of copper ions has been well established by a lot of articles. Ocean acidification, which increases the concentration of Cu^2+^, significantly increases the toxicity responses to mussels and purple sea urchins (Lewis et al., 2016). The correlation between the free copper concentration and bacterial growth seems to be better than the correlation involving the total concentration when the free copper concentration is seven to eight orders of magnitude lower than the total copper concentration (Hasman et al., 2009). Bopp et al. found that the reactive oxygen species formation rates were correlating with the free Cu^2+^ concentrations, which indicate the copper‐induced loss in viability and genotoxicity in trout gill cells are related to the free Cu^2+^ (Bopp & Abicht, 2008). Lorenzo et al. found the Cu^2+^, rather than the total copper concentrations, explained the toxicity of the Cu–humic acid solutions, and the Cu–humic acid complexes appeared as nontoxic forms. All these studies indicate that the toxic of copper ions cannot be ignored, despite the low content of it.

As various types of food may offer different content of copper ions in the digestive tract, we should pay attention to the toxicity of copper in different food. Besides, common food and drinking water are also a source of copper for human. With the widely use of copper plumbing, the concentration of copper in water may be high. It was found that the concentration of copper is 0.4–2.0 mg/L in copper pipes (Donohue et al., 2005; Lehtola et al., 2004; Pettersson & Rasmussen, 1999). As there are no organic ligands in drinking water, the concentration of copper ions in digestive tract may be high. In a study performed on rabbits, Sparks et al. found that the addition of trace amounts of copper (0.12 ppm) in water for 10 weeks induced cholesterol‐fed rabbits’ amyloid‐beta (Aβ) accumulation and significantly hindered the ability of rabbits to learn a difficult trace conditioning task (Sparks & Schreurs, 2003). For mice, a concentration of 0.13 mg/L Cu^2+^ in water increased Aβ production and neuroinflammation, increasing the severity of Alzheimer's disease (Singh et al., 2013). As more studies found the toxicity of copper in drinking water, the trace content of copper ions in digestive tract and their different toxicity should be future assessment.

## 5 CONCLUSION

As a transition metal element, copper is easy to bind to ligands because of its strong and nonspecific association with nearly all ligands, the coexisting components in the digestive tract can be seen as different ligands; the pH influences the acid–base dissociation of complexation group, which affects the concentration of the ligands; and the stability constants of copper complex decide the complexation strength with copper. In addition, molecule size and molecular steric also affect the interaction of copper and various ligands. As the environment in the digestive tract is complicated and changeable, it is hard to theoretically calculate or measure the accurate speciation of copper in digestive tract. However, different types of food will lead to different forms of copper distribution. The food rich in soluble ligands may lead to high soluble copper and then high bioavailability. And the food without ligands (water) may have high copper ions in digestive tract, which may lead to toxicity. As the widely use of copper plumbing, the safety of copper in drinking water should be widely concerned in the future.

## CONFLICT OF INTEREST

The authors have no conflict of interests.

## AUTHOR CONTRIBUTIONS

**Min Wu:** Data curation (equal); Formal analysis (equal); Funding acquisition (equal); Methodology (lead); Writing‐original draft (lead). **Leqin Ke:** Data curation (equal); Investigation (equal). **Mingyu Zhi:** Formal analysis (equal); Investigation (equal). **Yumei Qin:** Funding acquisition (equal); Project administration (lead); Resources (supporting); Writing‐review & editing (lead). **Jianzhong Han:** Project administration (supporting); Resources (lead); Writing‐review & editing (supporting).

## Data Availability

The data that support the findings of this study are available from the corresponding author upon reasonable request.
